# Lizard Brain: Tackling Locally Low-Dimensional Yet Globally Complex Organization of Multi-Dimensional Datasets

**DOI:** 10.3389/fnbot.2019.00110

**Published:** 2020-01-09

**Authors:** Jonathan Bac, Andrei Zinovyev

**Affiliations:** ^1^Institut Curie, PSL Research University, Paris, France; ^2^Institut National de la Santé et de la Recherche Médicale, U900, Paris, France; ^3^MINES ParisTech, CBIO-Centre for Computational Biology, PSL Research University, Paris, France; ^4^Centre de Recherches Interdisciplinaires, Université de Paris, Paris, France; ^5^Lobachevsky University, Nizhny Novgorod, Russia

**Keywords:** intrinsic dimension, dimension reduction, high-dimensional data, manifold learning, multi-manifold learning

## Abstract

Machine learning deals with datasets characterized by high dimensionality. However, in many cases, the intrinsic dimensionality of the datasets is surprisingly low. For example, the dimensionality of a robot's perception space can be large and multi-modal but its variables can have more or less complex non-linear interdependencies. Thus multidimensional data point clouds can be effectively located in the vicinity of principal varieties possessing locally small dimensionality, but having a globally complicated organization which is sometimes difficult to represent with regular mathematical objects (such as manifolds). We review modern machine learning approaches for extracting low-dimensional geometries from multi-dimensional data and their applications in various scientific fields.

## 1. Introduction : High-Dimensional Brain vs. Lizard Brain in High-Dimensional World

The space of robotic perception or human-robot-control interfaces formed by features extracted from raw sensor measurements (including self-perception recorded, for example, by force/torque sensors, and perception of other active players such as humans) is high-dimensional (multi-modal) and can be characterized by non-trivial geometry and topology (Artemiadis and Kyriakopoulos, 2010; Droniou et al., 2015). Planning and taking decisions requires active unsupervised learning of perception space structure and, if necessary, correction of the learnt models on the fly without destroying accumulated experience (Li et al., 2019). This might require the emergence of specialized functions in the robot “brain.”

Tackling the complexity of high-dimensional data spaces is a central challenge in machine learning. The famous notion of *curse of dimensionality* recapitulates difficulties with treating high-dimensional datasets, related to the mathematical theory of measure concentration (Giannopoulos and Milman, 2000; Gromov, 2003). In machine learning, among other manifestations it can refer to a distance measure's loss of discriminatory power as the intrinsic dimension of data increases, due to a concentration of pairwise distances between points toward the same mean value. In this setting, machine learning approaches which rely on the notion of neighboring data points perform badly. In practical applications, treating high-dimensional data can be challenging in terms of computational and memory demands. On the other hand, the curse can also be a blessing: essentially high-dimensional data point clouds possess surprisingly simple organization, which has been recently exploited in the framework of *high-dimensional brain in high-dimensional world* (Gorban et al., 2019b). High-dimensional brain is a model for the codification of memories composed from many sparsely connected neurons, each of which only deals with few high-dimensional data points, separating them from the rest of the data point cloud (Gorban et al., 2019b). It was applied to construct highly efficient error correctors of legacy AI systems, using non-iterative learning (Gorban et al., 2018).

The majority of unsupervised machine learning methods aim at reducing data's dimensionality or decomposing it into low-dimensional factors. This is opposite to the task of the high-dimensional brain, so we will call by analogy *lizard brain* a learning algorithm which is able to extract a useful low-dimensional representation of a high-dimensional data point cloud. Matching the level of data complexity, this representation can be complex and characterized by such features as non-linearity, discontinuity (e.g., coarse-grained clusters or other types of deviation from sampling independence and uniformity), bifurcations, non-trivial topologies and varying local intrinsic dimension (ID). By usefulness we mean that the extracted representation would improve downstream learning tasks; for example, by modifying point neighborhood relations and data space metrics. The name lizard brain is inspired by the triune brain theory, stating the existence of several layered mammalian brain substructures sequentially evolved and specialized in different types of animal behaviors (MacLean, 1990). We do not claim that the real reptilian brain or the reptilian complex is of low-dimensional nature: here we use this metaphor only to underline that an effective learning system should be composed of several parts, built on top of each other and dealing with opposite aspects of the high-dimensional world.

Distinct tasks of lizard and high-dimensional brains in machine learning reflect the complementarity principle (Gorban and Tyukin, 2018; Gorban et al., 2019a): the data space can be split into a low volume (low dimensional) subset, which requires nonlinear methods for constructing complex data approximators, and a high-dimensional subset, characterized by measure concentration, and simplicity allowing the effective application of linear methods. Machine learning methodology should suggest a method for making such splitting in real-life datasets, and propose tools specialized in dealing with intrinsically low- and high-dimensional data parts.

In this short review, we focus on methods for quantifying intrinsic dimensionality and constructing useful summaries of the data, by projection into low-dimensional space, or projection onto principal geometrical objects of lower complexity that approximate the structure of the data point cloud. We introduce a classification of these methods based on the notions of mathematical projection theory.

## 2. Defining and Measuring Intrinsic Dimension

The notion of *intrinsic dimension* (ID) intuitively refers to the minimal number of variables needed to represent data with little information loss. This concept, introduced in the field of signal analysis (Bennett, 1969), is largely used but doesn't have a consensus mathematical definition (Campadelli et al., 2015). In the context of the *manifold hypothesis*, i.e., when the data are considered to be a sample from an underlying *n*-dimensional manifold, the goal of ID estimation is to recover *n*.

Methods for ID estimation can be grouped by operating principle (Campadelli et al., 2015). The correlation dimension is an example of *fractal method* based on the fact that the number of points contained in a ball of growing radius *r* scales exponentially with the dimension of the underlying manifold (Grassberger and Procaccia, 1983). *Topological methods* estimate the topological dimension (e.g., as defined by the Lebesgue covering dimension) of a manifold. *Projective methods* look at the effect of mapping the points onto a lower-dimensional subspace, and set a threshold dimension based on a cost function and various heuristics (e.g., looking at variance gaps in the eigenspectra) (Fukunaga and Olsen, 1971; Bruske and Sommer, 1998; Little et al., 2009b; Fan et al., 2010). *Graph-based methods* exploit scaling properties of graphs, such as the length of the minimum spanning tree (Costa and Hero, 2004). *Nearest neighbors* methods rely on scaling properties of the distribution of local distances or angles, due for example to measure concentration (Levina and Bickel, 2004; Ceruti et al., 2014; Johnsson, 2016; Facco et al., 2017; Wissel, 2018; Amsaleg et al., 2019; Díaz et al., 2019; Gomtsyan et al., 2019). It has also been recently proposed to use the Fisher separability statistic (i.e., the probability of a data point to be separated from the rest of the data point cloud by a Fisher discriminant) for the estimation of ID (Gorban and Tyukin, 2018; Albergante et al., 2019). The observed distribution is compared in terms of this statistic to the one expected for i.i.d. samples from a uniform distribution of given dimension to find the one with closest properties (e.g., the distribution of the “equivalent sphere”).

Many ID estimators provide a single global ID value for the whole dataset but can be adapted to the case of varying local dimensionality by estimating the ID in data neighborhoods. The data contained in each neighborhood is usually assumed to be uniformly distributed over an *n*-dimensional ball (Levina and Bickel, 2004; Ceruti et al., 2014; Johnsson, 2016; Wissel, 2018; Díaz et al., 2019). In practice, ID proves sensitive to deviations from uniformity and neighborhood size (Little et al., 2009a; Campadelli et al., 2015). Benchmarks have shown that no single estimator today is ideal and using an ensemble of them is recommended (Campadelli et al., 2015; Camastra and Staiano, 2016).

## 3. Learning Low-Dimensional Structures of High-Dimensional Data Point Clouds

The task of the lizard brain is to learn the low-dimensional structure of a data point cloud *x*_*i*_, *i* = 1…*m*, existing in high-dimensional space *R*^*N*^. The principal mathematical approach to solve this task consists in defining a map (projection) ϕ from *R*^*N*^ to some base space *B* which is characterized by intrinsic dimension smaller than *N*. The large variety of algorithms learning low-dimensional data structures can be grouped with respect to the details of ϕ implementation and the structure of *B*. If *B* is Euclidean space *R*^*k*^, *k* << *N* then the approach is usually related to the *manifold learning* framework (Ma and Fu, 2011). However, *B* can be characterized by a more complex structure than simple Euclidean space: for example, it can have a non-trivial topology (of torus, sphere, dendroid, …). The base space can be discontinuous, such as a set of principal points learnt by *K*-means clustering. The algorithm can learn the base space structure as in the elastic principal graph method (Gorban et al., 2008b) or in the Growing Self-Organizing Maps (GSOM) (Alahakoon et al., 2000). Sometimes, these approaches are also named manifold learning techniques even though what is learnt can be more complex than a simple single manifold.

Below we classify a method by whether it assumes the base space *B* to be embedded or injected into the total space *R*^*N*^. In this case, we call a method *injective*, otherwise it is classified as *projective* (only the projection function to the base space is learnt). In the injective case, the base space *B* represents a subset of the initial data space *R*^*N*^. Typically, in injective methods we assume that the injected *B* is an approximation of data and use a nearest point for projection on *B*.

### 3.1. Injective Methods With Simple Euclidean Base Space

The classical method for extracting low-dimensional data structure is Principal Component Analysis (PCA) in which case *B* is simply a linear manifold in *R*^*N*^, ϕ is orthogonal projection onto *B*, and the sum of Euclidean distance squares $||x_{i}-\phi \left(x_{i}\right)|{|}^{2}$ is minimized (Jolliffe, 1993). Some non-linear extensions of PCA such as Hastie's principal curves (Hastie, 1984) or the piece-wise linear principal curves (Kégl and Krzyzak, 2002) are also injective methods as well as the popular Self-Organizing Map (SOM) (Kohonen, 1990). The SOM follows a stochastic approximation approach, while some of its descendant approaches optimize explicit functions: e.g., the Generative Topographic Map maximizes the likelihood of a low-dimensional Gaussian mixture distribution (Bishop et al., 1998), while the Elastic Map is based on optimization of the elastic energy functional (Gorban and Rossiev, 1999; Zinovyev, 2000; Gorban and Zinovyev, 2005, 2010; Gorban et al., 2008a), defined on a regular grid of nodes embedded into the data space. The Elastic Map approach can approximate data by manifolds with arbitrarily chosen topologies, e.g., by closed principal curves or spherical manifolds (Gorban and Zinovyev, 2005, 2009). For methods fitting a set of nodes to the data, the base space is either defined in the nodes of the grid or by linear interpolation between nodes: for example, a curve is defined as a set of nodes and linear segments connecting them, a 2D manifold is defined by triangulation of the grid and using linear segments, etc. The projection operator is frequently defined as a projection onto the nearest point of the manifold.

Currently we face a rapidly increasing interest in unsupervised learning methods based on artificial neural networks (ANNs). For example, the autoencoder ANNs, proposed in the early 90s, are trained to reproduce input data and are characterized by an hourglass organization, with a middle bottleneck layer containing few neurons and constraining the network to generate the output from a compressed input representation (Kramer, 1991; Hinton and Salakhutdinov, 2006). The base space is represented by the signals on the bottleneck layer neurons and usually is a simple Euclidean space. ANN-based autoencoders can be considered injective methods since any combination of signals at the bottleneck layer can be mapped back into the data space by the demapping ANN layers. Variational autoencoders learn in the bottleneck layer parameters of some intrinsically low-dimensional probabilistic graphical model generating the data (Kingma and Welling, 2013). Moreover, graph neural networks, including graph autoencoders, are able to perform dimensionality reduction by producing summarized graph-based embeddings of data (Scarselli et al., 2008), a feature related to the next section.

### 3.2. Injective Methods With Base Space Having Complex Structure

Injective methods with Euclidean base space help representing the intrinsic dataset complexity by reducing dimensionality but do not reflect this complexity in the structure of the base space. Other methods learn the structure of the base space such that it reflects that of the data point cloud. Initially (growing), neural gas algorithms used Hebbian learning to reconstruct summaries of data topology which can, however, remain too complex (Martinetz et al., 1991; Fritzke, 1995). The growing SOM derives regular base space structure which can have varying ID (Alahakoon et al., 2000).

Principal graphs together with methods for fitting them to data are a flexible framework for learning low-dimensional structures (Gorban and Zinovyev, 2010). In practice, the graph complexity should be constrained. For example, principal trees construct base spaces having dendroid topologies, which is achieved, in the Elastic Principal Graph (ElPiGraph) approach, by the application of topological grammar rules, transforming trees into trees and thus exploring only a space of trees (Gorban et al., 2007). A richer set of grammar rules can explore larger graph families (Albergante et al., 2018). Other methods are based on heuristics to guess the graph structure; for example, extracting the Minimal Spanning Tree from the kNN-graph in the Simple Principal Tree (simplePPT) method (Mao et al., 2015) automatically imposes the tree-like structure on the base space. Principal complexes combine the advantages of using regular grid (too restricted) and arbitrary graph (too complex) structures to approximate data. Here the graph grammar rules are applied to a small number of factor graphs, while the resulting structure of the approximating object is defined by the Cartesian product of factors (Gorban et al., 2007). For example, the Cartesian product of two linear graphs produces a 2D rectangular grid, and the Cartesian product of a tree-like graph with a linear graph will fit a branching sheet-like structure to the data. This approach allows constructing complex principal objects with ID larger than one controlling the complexity of graph factors only.

### 3.3. Projective Methods

In projective methods, the base space *B* which can possess more or less complex internal structure is not assumed to be a subset of the total space *R*^*N*^. This provides flexibility in the algorithm's construction but limits the capability for mapping new objects not participating in the definition of the projection (out-of-sample objects) from *R*^*N*^ into *B*. In other words, the mapping is learnt only for a subset of points in *B* corresponding to the data vectors $x_{i}\in R^{N}$ and not the rest of the data space. We note that the majority of projective methods start by computing an object similarity or dissimilarity matrix or offshoots of it, such as *k*-nearest neighbors (kNN) graph or ϵ-graph. The predecessor of many modern projective methods is the classical Multi-Dimensional Scaling (MDS) which is a linear projective alternative of PCA (Torgerson, 1952).

**Figure 1:**
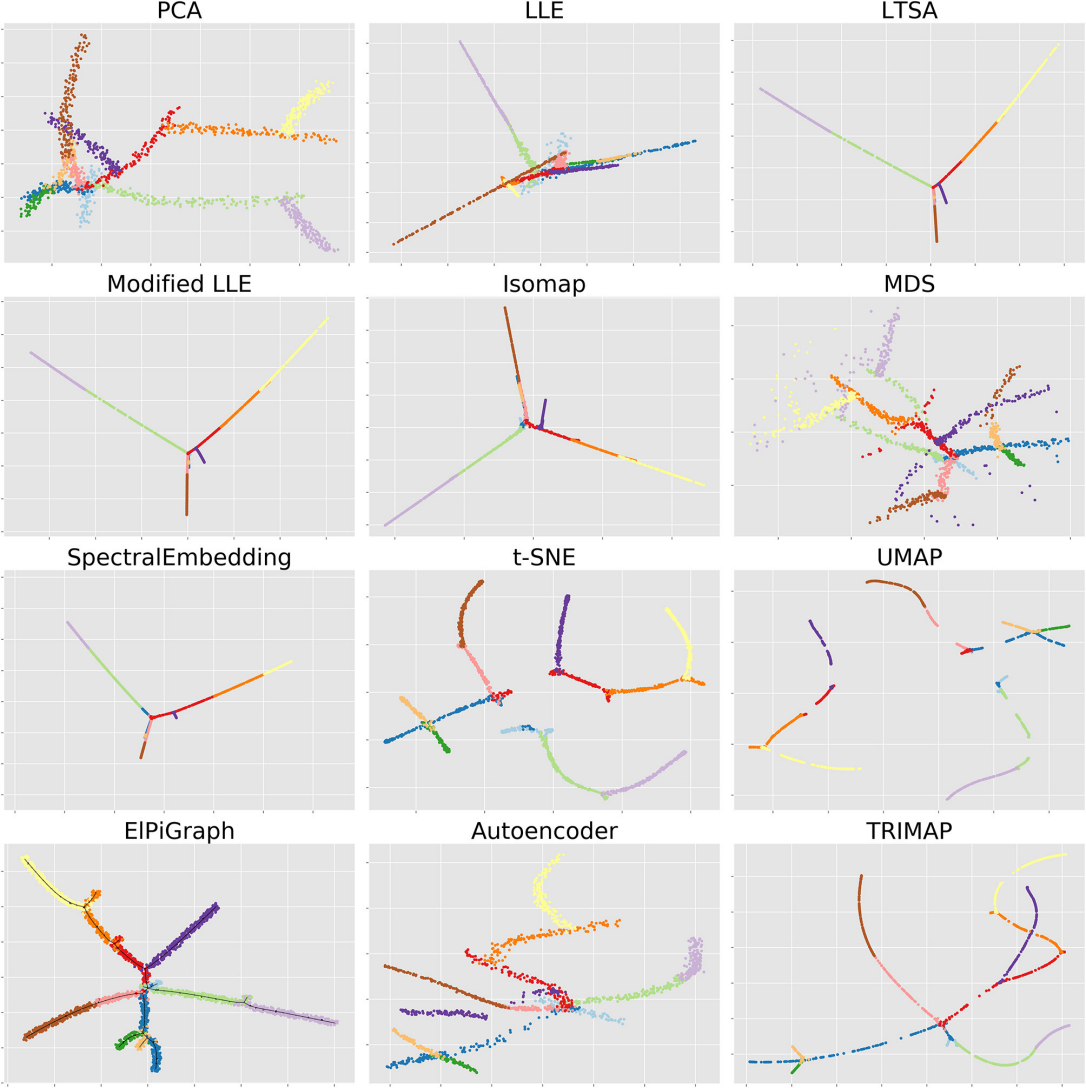
A simple inspiration example of a dataset, possessing low-dimensional intrinsic structure, which, however, remains hidden in any low-dimensional linear projection. The dataset is generated by a simple branching process, filling the volume of an *n*-dimensional hypercube: one starts with a non-linear (parabolic) trajectory from a random point inside the cube which goes up to one of its faces. Then it stops, a random point from the previously generated points is selected, and a new non-linear trajectory starts in a random direction. The process continues to generate *k* branches; then the data point cloud is generated by adding a uniformly distributed noise around the generated trajectories. If *k* is large enough then the global estimate of the dataset dimensionality will be close to *n*: however, the local intrinsic dimension of the dataset remains one (or, between one and two, in the vicinity of branch junctions or intersections). The task of the lizard brain is to uncover the low-dimensional structure of this dataset: in particular, classify the data points into the underlying trajectory branches and uncover the tree-like structure of branch connections. The figure shows how various unsupervised machine learning methods mentioned in this review capture the complexity of this dataset having only *k* = 12 branches generated with *n* = 10 (each shown in color) in 2D projections. Most of the methods here use simple Euclidean base space, besides ElPiGraph, in which case the structure of the base space (tree-like) is shown by a black line and the 2D representation is created by using the force-directed layout of the graph.

The most popular representatives of non-linear projective methods are ISOMAP (Tenenbaum et al., 2000), Laplacian and Hessian Eigenmaps (Belkin and Niyogi, 2003; Donoho and Grimes, 2003) and Diffusion maps (Coifman and Lafon, 2006), in which the main idea is to define object dissimilarity reflecting the geodesic distances along the kNN- or ϵ-graph (see Figure 1). Local Linear Embedding (LLE) aims at reproducing, in the low-dimensional space, local linear relations between objects in the total space and assemble them into a global picture (Roweis and Saul, 2000; Zhang and Wang, 2007). Kernel PCA exploits the kernel trick and applies MDS on a kernel-modified Gram matrix (Schölkopf et al., 1998; Bengio et al., 2004a; Ham et al., 2004). On top of the original formulations, many generalizations of these methods have been produced recently. For example, the vector diffusion map (Singer and Wu, 2012) doesn't use operators on the manifold itself but differential operators on fiber bundles over the manifold. Grassmann&Stiefel Eigenmaps require proximity between the original manifold and its estimator but also between their tangent spaces (Bernstein and Kuleshov, 2012; Bernstein et al., 2015). The limitations of the projective methods are partially overcome in some of their out-of-sample extensions that allow the mapping of new points without having to recompute eigenvectors (Bengio et al., 2004b; Qiao et al., 2012).

Several methods for projective dimensionality reduction, such as t-distributed stochastic neighboring embedding (t-SNE) (Maaten and Hinton, 2008) or more recent Uniform Manifold Approximation and Projection (UMAP) (McInnes et al., 2018) found overwhelming number of applications in applied data science, e.g., for visualizing large-scale molecular profiling data in biology. One of the reasons for their popularity is their focus on more accurate representation of small distances (rather than large ones as in PCA) between data vectors, which frequently match better the purpose of data visualization/representation.

Projective methods are extremely popular in modern machine learning for non-linear dimensionality reduction, and new ideas are constantly explored: here we can mention kernel density estimation (Mohammed and Narayanan, 2017), genetic programming (Lensen et al., 2019), parallel transport (Budninskiy et al., 2019), triplet information (TRIMAP) (Amid and Warmuth, 2019).

While the vast majority of methods use projection onto Euclidean base space, some authors have also suggested the use of classical algorithms for non-Euclidean embeddings, such as hyperbolic or spherical spaces (Begelfor and Werman, 2005; Cvetkovski and Crovella, 2017). Recently, several works have shown benefits of non-Euclidean embeddings for the particular case of graph data, which can have intrinsic curvature (Walter and Ritter, 2002; Chamberlain et al., 2017; Muscoloni et al., 2017; Nickel and Kiela, 2017).

### 3.4. Multi-Manifold and Manifold Alignment Learning

The complex and sometimes discontinuous organization of real-life data can be a challenge for the single manifold hypothesis, which underlies many algorithms. In some cases, data is better described as sampled from multiple manifolds. For example, the task of face recognition can be described by the identification of different manifolds, each corresponding to a different person's facial images (Yang et al., 2007). Another example is LIDAR technology, which generates 3D point clouds in the form of the surrounding terrain (e.g., a bridge will result in a flat 2D surface for the road, 1D cables, etc.) (Medina et al., 2019).

The existence of such data motivates approaches that account for the presence of multiple and potentially intersecting manifolds. A first idea to deal with such scenario is to measure local ID to identify structures with variable ID in a dataset. As a natural next step, the data can be segmented accordingly to the local ID (see Allegra et al., 2019 and references therein). Beyond such segmentation, one can integrate classical algorithms into a complete framework to perform the detection and reconstruction of manifold structures. Such frameworks have been recently introduced based on well-known algorithms, such as spectral clustering and local tangent space estimation (Wang et al., 2010, 2011; Gong et al., 2012), LLE (Hettiarachchi and Peters, 2015), ISOMAP (Fan et al., 2012; Yang et al., 2016; Li et al., 2017; Mahapatra and Chandola, 2017) and local PCA (Arias-Castro et al., 2017). Other approaches use less classical techniques such as tensor voting (Mordohai and Medioni, 2010; Deutsch and Medioni, 2015, 2016), variational autoencoders (Ye and Zhao, 2019), or multi-agent flow (Shen and Han, 2016).

Another task which becomes important in some scientific domains is to learn distinct maps from several data spaces to the common base space. The general idea here is to align, according to some criteria, multiple projections of the data point clouds; therefore, this family of methods is sometimes termed “manifold alignment” (Ma and Fu, 2011). Details of the problem formulation are important here and can constrain the method applicability. For example, Generalized Unsupervised Manifold Alignment (GUMA) assumes a possibility of one-to-one mapping between two data spaces (Cui et al., 2014). The Manifold Alignment Generative Adversarial Network (MAGAN) uses generative adversarial networks (GAN) to use one data space as a base space for a second data space, and vice versa (Amodio and Krishnaswamy, 2018); it assumes either some shared variables or partly matched pairs of points between two data spaces.

## 4. Discussion

In this short review we highlight that many globally multi-dimensional datasets used in the field of machine learning and artificial intelligence can possess intrinsically low-dimensional structure, which yet can be highly complex. The task of a lizard brain (methaphoric opposite to the high-dimensional brain, composed of sparsely connected concept neurons) is to detect which parts of the data are essentially low-dimensional and to extract the low-dimensional structure from high-dimensional space. Well-established manifold learning frameworks can be used for this purpose, taking into account some recent developments mentioned above. At the same time, new approaches learning structures more general than simple connected manifolds are needed in concrete applications. Thus, the structure of real-life datasets can be characterized by strong noise, bifurcation-like patterns, self-intersecting flows, variable local ID, fine-grained lumping, and other features not easily captured by the manifold-type objects. There exists candidate methodologies such as data approximation by principal cubic complexes, using topological grammar approach, which can overcome some limitations of the simple manifold-based approaches.

There are scientific fields where the data possessing complex yet locally low-dimensional structure are generated at large scale. One example of this is molecular profiling of single cells in molecular biology, where the generated clouds of data points are characterized by many of the above mentioned complex features. Today we face a boom of machine learning-based methodology development aiming at treating this data type (Chen et al., 2019; Saelens et al., 2019). Another well-known example is reconstructing the surrounding environment from point clouds generated by LIDAR technology.

Further efforts are needed to supply the lizard brain with algorithmic approaches suitable in the various contexts of real-life data. The development of benchmark datasets and new benchmarking methodologies is also needed to assess the efficiency and applicability of the existing toolbox for extracting low-dimensional structures from high-dimensional data.

## Author Contributions

AZ and JB jointly defined the scope of the review, its bibliography and classification of methods, wrote the review and together worked on the implementation of the Jupyter notebook.

### Conflict of Interest

The authors declare that the research was conducted in the absence of any commercial or financial relationships that could be construed as a potential conflict of interest.

## References

[B1] Alahakoon D. Halgamuge S. K. Srinivasan B. (2000). Dynamic self-organizing maps with controlled growth for knowledge discovery. IEEE Trans. Neural Netw. 11, 601–614. 10.1109/72.846732 18249788

[B2] Albergante L. Bac J. Zinovyev A. (2019). Estimating the effective dimension of large biological datasets using fisher separability analysis, in Proceedings of the IEEE IJCNN 2019 - International Joint Conference on Neural Networks (Budapest: IEEE). 10.1109/IJCNN.2019.8852450

[B3] Albergante L. Mirkes E. M. Chen H. Martin A. Faure L. Barillot E. (2018). Robust and scalable learning of complex dataset topologies via ElPiGraph. arXiv [preprint] arXiv:1804.07580. 10.3390/e22030296 PMC7516753 33286070

[B4] Allegra M. Facco E. Laio A. Mira A. (2019). Clustering by the local intrinsic dimension: the hidden structure of real-world data. arXiv [preprint] arXiv:1902.10459.

[B5] Amid E. Warmuth M. K. (2019). Trimap: large-scale dimensionality reduction using triplets. arXiv [preprint] arXiv:1910.00204.

[B6] Amodio M. Krishnaswamy S. (2018). MAGAN: aligning biological manifolds. arXiv [preprint] arXiv:1803.00385.

[B7] Amsaleg L. Chelly O. Houle M. E. Kawarabayashi K.-I. Radovanović M. Treeratanajaru W. (2019). Intrinsic dimensionality estimation within tight localities, in Proceedings of the 2019 SIAM International Conference on Data Mining (SIAM), 181–189.

[B8] Arias-Castro E. Lerman G. Zhang T. (2017). Spectral clustering based on local PCA. J. Mach. Learn. Res. 18, 253–309. Available online at: http://dl.acm.org/citation.cfm?id=3122009.3122018

[B9] Artemiadis P. K. Kyriakopoulos K. J. (2010). EMG-based control of a robot arm using low-dimensional embeddings. IEEE Trans. Robot. 26, 393–398. 10.1109/TRO.2009.2039378

[B10] Begelfor E. Werman M. (2005). The world is not always flat or learning curved manifolds. School Eng. Comput. Sci. Hebrew Univer. Jerusalem. Tech. Rep. 3:8 Available online at: http://www.cs.huji.ac.il/~werman/Papers/cmds.pdf

[B11] Belkin M. Niyogi P. (2003). Laplacian eigenmaps for dimensionality reduction and data representation. Neural Comput. 15, 1373–1396. 10.1162/089976603321780317

[B12] Bengio Y. Delalleau O. Roux N. L. Paiement J.-F. Vincent P. Ouimet M. (2004a). Learning eigenfunctions links spectral embedding and kernel PCA. Neural Comput. (Montreal, QC) 16, 2197–2219. 10.1162/0899766041732396 15333211

[B13] Bengio Y. Paiement J.-F. Vincent P. Delalleau O. Roux N. L. Ouimet M. (2004b). Out-of-sample extensions for LLE, ISOMAP, MDS, eigenmaps, and spectral clustering, in Advances in Neural Information Processing Systems, 177–184.

[B14] Bennett R. (1969). The intrinsic dimensionality of signal collections. IEEE Trans. Inform. Theory 15, 517–525.

[B15] Bernstein A. Kuleshov A. Yanovich Y. (2015). Information preserving and locally isometric&conformal embedding via tangent manifold learning, in 2015 IEEE International Conference on Data Science and Advanced Analytics (DSAA) (Paris: IEEE), 1–9. 10.1109/DSAA.2015.7344815

[B16] Bernstein A. V. Kuleshov A. P. (2012). Tangent bundle manifold learning via Grassmann&Stiefel eigenmaps. arXiv [preprint] arXiv:1212.6031.

[B17] Bishop C. M. Svensén M. Williams C. K. (1998). GTM: the generative topographic mapping. Neural Comput. 10, 215–234.

[B18] Bruske J. Sommer G. (1998). Intrinsic dimensionality estimation with optimally topology preserving maps. IEEE Trans. Pattern Analy. Mach. Intell. 20, 572–575. 10.1109/34.682189

[B19] Budninskiy M. Yin G. Feng L. Tong Y. Desbrun M. (2019). Parallel transport unfolding: a connection-based manifold learning approach. SIAM J. Appl. Algebra Geomet. 3, 266–291. 10.1137/18M1196133

[B20] Camastra F. Staiano A. (2016). Intrinsic dimension estimation: advances and open problems. Inform. Sci. 328, 26–41. 10.1016/j.ins.2015.08.029

[B21] Campadelli P. Casiraghi E. Ceruti C. Rozza A. (2015). Intrinsic dimension estimation: relevant techniques and a benchmark framework. Math. Prob. Eng. 2015, 1–21. 10.1155/2015/759567

[B22] Ceruti C. Bassis S. Rozza A. Lombardi G. Casiraghi E. Campadelli P. (2014). DANCo: An intrinsic dimensionality estimator exploiting angle and norm concentration. Pattern Recognit. 47, 2569–2581. 10.1016/j.patcog.2014.02.013

[B23] Chamberlain B. P. Clough J. Deisenroth M. P. (2017). Neural embeddings of graphs in hyperbolic space. arXiv [preprint] arXiv:1705.10359.

[B24] Chen H. Albergante L. Hsu J. Y. Lareau C. A. Lo Bosco G. Guan J.. (2019). Single-cell trajectories reconstruction, exploration and mapping of omics data with STREAM. Nat. Commun. 10:1903. 10.1038/s41467-019-09670-4 31015418 PMC6478907

[B25] Coifman R. R. Lafon S. (2006). Diffusion maps. Appl. Comput. Harmon. Analy. 21, 5–30. 10.1016/j.acha.2006.04.006

[B26] Costa J. A. Hero A. O. (2004). Geodesic entropic graphs for dimension and entropy estimation in manifold learning. IEEE Trans. Signal Process. 52, 2210–2221. 10.1109/TSP.2004.831130

[B27] Cui Z. Chang H. Shan S. Chen X. (2014). Generalized unsupervised manifold alignment, in Advances in Neural Information Processing Systems, (Montreal, QC) 2429–2437.

[B28] Cvetkovski A. Crovella M. (2017). Low-stress data embedding in the hyperbolic plane using multidimensional scaling. Appl. Math. 11, 5–12. 10.18576/amis/110102

[B29] Deutsch S. Medioni G. (2016). Learning the geometric structure of manifolds with singularities using the tensor voting graph. J. Math. Imaging. Vision 57, 402–422. 10.1007/s10851-016-0684-2

[B30] Deutsch S. Medioni G. G. (2015). Intersecting manifolds: detection, segmentation, and labeling, in Twenty-Fourth International Joint Conference on Artificial Intelligence, (Buenos Aires) 3445–3452.

[B31] Díaz M. Quiroz A. J. Velasco M. (2019). Local angles and dimension estimation from data on manifolds. J. Multivar. Analy. 173, 229–247. 10.1016/j.jmva.2019.02.014

[B32] Donoho D. L. Grimes C. (2003). Hessian eigenmaps: locally linear embedding techniques for high-dimensional data. Proc. Natl. Acad. Sci. U.S.A. 100, 5591–5596. 10.1073/pnas.1031596100 16576753 PMC156245

[B33] Droniou A. Ivaldi S. Sigaud O. (2015). Deep unsupervised network for multimodal perception, representation and classification. Robot. Autonomous Syst. 71, 83–98. 10.1016/j.robot.2014.11.005

[B34] Facco E. D'Errico M. Rodriguez A. Laio A. (2017). Estimating the intrinsic dimension of datasets by a minimal neighborhood information. Sci. Rep. 7:12140. 10.1038/s41598-017-11873-y 28939866 PMC5610237

[B35] Fan M. Gu N. Qiao H. Zhang B. (2010). Intrinsic dimension estimation of data by principal component analysis. arXiv 1002.2050 [cs.CV].

[B36] Fan M. Qiao H. Zhang B. Zhang X. (2012). Isometric multi-manifold learning for feature extraction in 2012 IEEE 12th International Conference on Data Mining (Brussels: IEEE) 241–250. 10.1109/ICDM.2012.98

[B37] Fritzke B. (1995). A growing neural gas network learns topologies, in Advances in Neural Information Processing Systems, (Denver, CO) 625–632.

[B38] Fukunaga K. Olsen D. (1971). An algorithm for finding intrinsic dimensionality of data. IEEE Trans. Comput. C-20, 176–183.

[B39] Giannopoulos A. Milman V. (2000). Concentration property on probability spaces. Adv. Math. 156, 77–106. 10.1006/aima.2000.1949

[B40] Gomtsyan M. Mokrov N. Panov M. Yanovich Y. (2019). Geometry-aware maximum likelihood estimation of intrinsic dimension. arXiv [preprint] arXiv:1904.06151.

[B41] Gong D. Zhao X. Medioni G. (2012). Robust multiple manifolds structure learning. arXiv [preprint] arXiv:1206.4624.

[B42] Gorban A. Golubkov A. Grechuk B. Mirkes E. Tyukin I. (2018). Correction of AI systems by linear discriminants: probabilistic foundations. Inf. Sci. (New York, NY) 466, 303–322. 10.1016/j.ins.2018.07.040

[B43] Gorban A. Kégl B. Wunsch D. Zinovyev A. editors (2008a). Principal Manifolds for Data Visualisation and Dimension Reduction. Berlin; Heidelberg; New York, NY: Springer.

[B44] Gorban A. Makarov V. Tyukin I. (2019a). Symphony of high-dimensional brain. Reply to comments on “The unreasonable effectiveness of small neural ensembles in high-dimensional brain”. Phys. Life Rev. 29, 115–119. 10.1016/j.plrev.2019.06.003 31272910

[B45] Gorban A. Makarov V. Tyukin I. (2019b). The unreasonable effectiveness of small neural ensembles in high-dimensional brain. Phys. Life Rev. 29, 55–88. 10.1016/j.plrev.2018.09.005 30366739

[B46] Gorban A. Rossiev A. A. (1999). Neural network iterative method of principal curves for data with gaps. J. Comput. Syst. Sci. Int. 38, 825–830.

[B47] Gorban A. Tyukin I. (2018). Blessing of dimensionality: mathematical foundations of the statistical physics of data. Phil. Trans. R. Soc. A 376:20170237. 10.1098/rsta.2017.0237 29555807 PMC5869543

[B48] Gorban A. Zinovyev A. (2005). Elastic principal graphs and manifolds and their practical applications. Computing 75, 359–379. 10.1007/s00607-005-0122-6

[B49] Gorban A. Zinovyev A. (2010). Principal manifolds and graphs in practice: from molecular biology to dynamical systems. Int. J. Neural Syst. 20, 219–232. 10.1142/S0129065710002383 20556849

[B50] Gorban A. N. Sumner N. R. Zinovyev A. Y. (2007). Topological grammars for data approximation. Appl. Math. Lett. 20, 382–386. 10.1016/j.aml.2006.04.022

[B51] Gorban A. N. Sumner N. R. Zinovyev A. Y. (2008b). Beyond the concept of manifolds: Principal trees, metro maps, and elastic cubic complexes, in Principal Manifolds for Data Visualization and Dimension Reduction (Springer), 219–237.

[B52] Gorban A. N. Zinovyev A. (2009). Principal graphs and manifolds, in Handbook of Research on Machine Learning Applications and Trends: Algorithms, Methods and Techniques, eds Olivas E. S. Guererro J. D. M. Sober M. M. Benedito J. R. M. Lopes A.J.S.

[B53] Grassberger P. Procaccia I. (1983). Measuring the strangeness of strange attractors. Phys. D Nonlinear Phenom. 9, 189–208.

[B54] Gromov M. (2003). Isoperimetry of waists and concentration of maps. Geom. Funct. Anal. 13, 178–215. 10.1007/s000390300004

[B55] Ham J. H. Lee D. D. Mika S. Schölkopf B. (2004). A kernel view of the dimensionality reduction of manifolds. Departmental Papers (ESE), (Philadelphia, PA) 93.

[B56] Hastie T. (1984). Principal curves and surfaces. Technical report, Stanford university, CA, Lab for computational statistics.

[B57] Hettiarachchi R. Peters J. F. (2015). Multi-manifold LLE learning in pattern recognition. Patt. Recognit. 48, 2947–2960. 10.1016/j.patcog.2015.04.003

[B58] Hinton G. E. Salakhutdinov R. R. (2006). Reducing the dimensionality of data with neural networks. Science 313, 504–507. 10.1126/science.1127647 16873662

[B59] Johnsson K. (2016). Structures in High-Dimensional Data: Intrinsic Dimension and Cluster Analysis. Ph.D. thesis, Faculty of Engineering, LTH.

[B60] Jolliffe I. (1993). Principal Component Analysis. Berlin; Heidelberg: Springer.

[B61] Kégl B. Krzyzak A. (2002). Piecewise linear skeletonization using principal curves. IEEE Trans. Patt. Analy. Mach. Intell. 24, 59–74. 10.1109/34.982884

[B62] Kingma D. P. Welling M. (2013). Auto-encoding variational bayes. arXiv [preprint] arXiv:1312.6114.

[B63] Kohonen T. (1990). The self-organizing map. Proc. IEEE 78, 1464–1480.

[B64] Kramer M. A. (1991). Nonlinear principal component analysis using autoassociative neural networks. AIChE J. 37, 233–243.

[B65] Lensen A. Xue B. Zhang M. (2019). Can genetic programming do manifold learning too? in European Conference on Genetic Programming (Leipzig: Springer), 114–130.

[B66] Levina E. Bickel P. J. (2004). Maximum Likelihood estimation of intrinsic dimension, in Proceedings of the 17th International Conference on Neural Information Processing Systems (Vancouver, BC: MIT Press), 777–784.

[B67] Li X. Cai C. He J. (2017). Density-based multi-manifold ISOMAP for data classification, in 2017 Asia-Pacific Signal and Information Processing Association Annual Summit and Conference (APSIPA ASC) (Kuala Lumpur: IEEE), 897–903.

[B68] Li Z. Chen F. Bicchi A. Sun Y. Fukuda T. (2019). Guest Editorial Neuro-Robotics Systems: Sensing, Cognition, Learning, and Control. IEEE Trans. Cogn. Dev. Syst. 11, 145–147. 10.1109/TCDS.2019.2915408

[B69] Little A. V. Jung Y.-M. Maggioni M. (2009a). Multiscale Estimation of Intrinsic Dimensionality of Data Sets. Technical report. Arlington, TX, United States.

[B70] Little A. V. Lee J. Jung Y.-M. Maggioni M. (2009b). Estimation of intrinsic dimensionality of samples from noisy low-dimensional manifolds in high dimensions with multiscale SVD, in 2009 IEEE/SP 15th Workshop on Statistical Signal Processing (Cardiff: IEEE) 85–88.

[B71] Ma Y. Fu Y. (2011). Manifold Learning Theory and Applications. Boca Raton, FL: CRC press.

[B72] Maaten L. v. d. Hinton G. (2008). Visualizing data using t-SNE. J. Mach. Learn. Res. 9, 2579–2605. Available online at: http://www.jmlr.org/papers/v9/vandermaaten08a.html

[B73] MacLean P. D. (1990). The Triune Brain in Evolution. New York, NY: Plenum.

[B74] Mahapatra S. Chandola V. (2017). S-isomap++: multi manifold learning from streaming data, in IEEE International Conference on Big Data (Big Data) (Boston, MA: IEEE), 716–725.

[B75] Mao Q. Yang L. Wang L. Goodison S. Sun Y. (2015). SimplePPT: a simple principal tree algorithm, in Proceedings of the 2015 SIAM International Conference on Data Mining (Vancouver, BC: SIAM), 792–800.

[B76] Martinetz T. Schulten K. (1991). A “neural-gas” Network Learns Topologies. Champaign, IL: University of Illinois at Urbana-Champaign.

[B77] McInnes L. Healy J. Melville J. (2018). UMAP: Uniform manifold approximation and projection for dimension reduction. arXiv [preprint] arXiv:1802.03426.

[B78] Medina F. P. Ness L. Weber M. Djima K. Y. (2019). Heuristic framework for multiscale testing of the multi-manifold hypothesis, in Research in Data Science, eds Gasparovic E. Domeniconi C. (Providence, RI: Springer), 47–80.

[B79] Mohammed K. Narayanan H. (2017). Manifold learning using kernel density estimation and local principal components analysis. arXiv [preprint] arXiv:1709.03615.

[B80] Mordohai P. Medioni G. (2010). Dimensionality estimation, manifold learning and function approximation using tensor voting. J. Mach. Learn. Res. 11, 411–450.

[B81] Muscoloni A. Thomas J. M. Ciucci S. Bianconi G. Cannistraci C. V. (2017). Machine learning meets complex networks via coalescent embedding in the hyperbolic space. Nat. Commun. 8:1615. 10.1038/s41467-017-01825-5 29151574 PMC5694768

[B82] Nickel M. Kiela D. (2017). Poincaré embeddings for learning hierarchical representations, in Advances in Neural Information Processing Systems, (Long Beach, CA) 6338–6347.

[B83] Qiao H. Zhang P. Wang D. Zhang B. (2012). An explicit nonlinear mapping for manifold learning. IEEE Trans. Cybernet. 43, 51–63. 10.1109/TSMCB.2012.2198916 22736649

[B84] Roweis S. T. Saul L. K. (2000). Nonlinear dimensionality reduction by locally linear embedding. Science 290, 2323–2326. 10.1126/science.290.5500.2323 11125150

[B85] Saelens W. Cannoodt R. Todorov H. Saeys Y. (2019). A comparison of single-cell trajectory inference methods. Nat. Biotechnol. 37, 547–554. 10.1038/s41587-019-0071-9 30936559

[B86] Scarselli F. Gori M. Tsoi A. C. Hagenbuchner M. Monfardini G. (2008). The graph neural network model. IEEE Trans. Neural Netw. 20, 61–80. 10.1109/TNN.2008.2005605 19068426

[B87] Schölkopf B. Smola A. Müller K.-R. (1998). Nonlinear component analysis as a kernel eigenvalue problem. Neural Comput. 10, 1299–1319.

[B88] Shen G. Han D. (2016). A flow based approach for learning multiple manifolds, in 9th International Congress on Image and Signal Processing, BioMedical Engineering and Informatics (CISP-BMEI) (Datong: IEEE), 1905–1910.

[B89] Singer A. Wu H.-T. (2012). Vector diffusion maps and the connection Laplacian. Commun. Pure Appl. Mathemat. 65, 1067–1144. 10.1002/cpa.21395 24415793 PMC3886882

[B90] Tenenbaum J. B. Silva V. d. Langford J. C. (2000). A global geometric framework for nonlinear dimensionality reduction. Science 290, 2319–2323. 10.1126/science.290.5500.2319 11125149

[B91] Torgerson W. S. (1952). Multidimensional scaling: I. theory and method. Psychometrika 17, 401–419. 10.1007/BF02289530 5217606

[B92] Walter J. A. Ritter H. (2002). On interactive visualization of high-dimensional data using the hyperbolic plane, in Proceedings of the Eighth ACM SIGKDD International Conference on Knowledge Discovery and Data Mining (New York, NY: ACM), 123–132.

[B93] Wang Y. Jiang Y. Wu Y. Zhou Z.-H. (2010). Multi-manifold clustering, in Pacific Rim International Conference on Artificial Intelligence, (Daegu) 280–291.

[B94] Wang Y. Jiang Y. Wu Y. Zhou Z.-H. (2011). Spectral clustering on multiple manifolds. IEEE Trans. Neural Netw. 22, 1149–1161. 10.1109/TNN.2011.2147798 21690009

[B95] Wissel D. R. (2018). Intrinsic Dimension Estimation using Simplex Volumes. Ph.D. thesis, University of Bonn, Bonn, Germany.

[B96] Yang B. Xiang M. Zhang Y. (2016). Multi-manifold discriminant Isomap for visualization and classification. Patt. Recog. 55, 215–230. 10.1016/j.patcog.2016.02.001

[B97] Yang J. Zhang D. Yang J.-y. Niu B. (2007). Globally maximizing, locally minimizing: unsupervised discriminant projection with applications to face and palm biometrics. IEEE Trans. Patt. Analy. Mach. Intell. 29, 650–664. 10.1109/TPAMI.2007.1008 17299222

[B98] Ye X. Zhao J. (2019). Multi-manifold clustering: a graph-constrained deep nonparametric method. Patt. Recogn. 93, 215–227. 10.1016/j.patcog.2019.04.029

[B99] Zhang Z. Wang J. (2007). MLLE: modified locally linear embedding using multiple weights, in Advances in Neural Information Processing Systems, (Vancouver, BC) 1593–1600.

[B100] Zinovyev A. (2000). Visualization of Multidimensional Data [in Russian]. Krasnoyarsk: Krasnoyarsk State Technical University Press.

